# Outcome measures for assessing the effectiveness of physiotherapy interventions on equinus foot deformity in post-stroke patients with triceps surae spasticity: A scoping review

**DOI:** 10.1371/journal.pone.0287220

**Published:** 2023-10-12

**Authors:** Isabella Campanini, Maria Chiara Bò, Maria Chiara Bassi, Benedetta Damiano, Sara Scaltriti, Mirco Lusuardi, Andrea Merlo

**Affiliations:** 1 Neuromotor and Rehabilitation Department, LAM–Motion Analysis Laboratory, San Sebastiano Hospital, Azienda USL-IRCCS di Reggio Emilia, Correggio (Reggio Emilia), Correggio, Italy; 2 Merlo Bioengineering, Parma, Italy; 3 Medical Library, Azienda USL-IRCCS di Reggio Emilia, Correggio, Italy; 4 Neuromotor and Rehabilitation Department, Azienda USL-IRCCS Reggio Emilia, Correggio, Italy; Western Michigan University, UNITED STATES

## Abstract

**Objective:**

Equinus foot deformity (EFD) is the most common deviation after stroke. Several physiotherapy interventions have been suggested to treat it. However, studies evaluating the efficacy of these treatments vary widely in terms of assessment modalities, type of data analysis, and nomenclature. This scoping review aimed to map current available evidence on outcome measures and the modalities employed to assess the effectiveness of physiotherapy programs for the reduction of triceps surae (TS) spasticity and EFD in patients with stroke.

**Methods:**

Scoping review methodological frameworks have been used. Three databases were investigated. Primary literature addressing TS spasticity in adult patients with stroke using physiotherapy interventions was included. Findings were systematically summarized in tables according to the intervention used, intervention dosage, control group, clinical, and instrumental outcome measures.

**Results:**

Of the 642 retrieved studies, 53 papers were included. TS spasticity was assessed by manual maneuvers performed by clinicians (mainly using the Ashworth Scale), functional tests, mechanical evaluation through robotic devices, or instrumental analysis and imaging (such as the torque-angle ratio, the H-reflex, and ultrasound images). A thorough critical appraisal of the construct validity of the scales and of the statistics employed was provided, particularly focusing on the choice of parametric and non-parametric approaches when using ordinal scales. Finally, the complexity surrounding the concept of “spasticity” and the possibility of assessing the several underlying active and passive causes of EFD, with a consequent bespoke treatment for each of them, was discussed.

**Conclusion:**

This scoping review provides a comprehensive description of all outcome measures and assessment modalities used in literature to assess the effectiveness of physiotherapy treatments, when used for the reduction of TS spasticity and EFD in patients with stroke. Clinicians and researchers can find an easy-to-consult summary that can support both their clinical and research activities.

## 1 Introduction

In adult patients, the equinus foot deviation (EFD) is one of the most common acquired deformities of the lower limb following a stroke. The ankle is positioned in a plantarflexed stance, and it is also usually associated with supination of the foot leading to the equinovarus deviation (EVFD). Clawed toes can also be present [1]. EFD could be due to multiple factors, including paresis of the dorsiflexor muscles, plantarflexor muscles overactivity, stiffness, viscosity and contracture [1].

Cerebrovascular accidents can have several sequelae involving different functions. They often lead to cognitive impairments, sensory loss, difficulty in isolating movements, and increased muscle tone with the onset of pathological synergies [2]. EFD has considerable impacts on gait, balance, and safety. EFD is the most frequent acquired lower limb deformity in the population with stroke, and directly affects walking ability. It might alter both the stability of the foot-ankle complex during the stance phase of gait and the ability of foot clearance during the swing phase [3]. Given the resulting pain, instability, and increased risk of falls, patients often need supervision and different levels of assistance during their outings, depending on the degree of the impairment. This reduces the odds of a successful return to social and working activities and requires an outflow of resources to remain in the community [4], affecting patients’ and caregivers’ quality of life [5,6] and increasing the economic burden for health care systems [7].

In literature, several treatments have been suggested for the management of spasticity-related EFD in patients with stroke. Conservative interventions include botulinum toxin injections, focal inhibition, oral or intrathecal medications, serial casting or orthosis, and physiotherapy (PT) [8]. Surgery is performed by neuro-orthopedic surgeons when chronic deformities develop [3,9].

Recent studies have focused on many PT treatments of triceps surae (TS) spasticity following stroke [5,8,10–14]. These include stretching, shock waves, electrostimulation, dry-needling, transcutaneous electrical nerve stimulation, vibrations, ultrasound, cryotherapy, and physiotherapist-guided physical exercising. On the one hand, some of these treatments, such as shock waves, dry needling, and electrical stimulation, showed similar promising results. On the other hand, when analyzing these studies, at least two weak points can be identified [14]. Firstly, the countless modalities used for the assessment of spasticity in EVFD prevent direct comparisons of the results. Secondly, the type of statistical analysis chosen is often incorrect when dealing with clinical scales, since parametric statistics (e.g., mean value and t-test) cannot be used when analyzing ordinal scores.

Given the variety of outcome measures and data analysis procedures used in the studies on PT interventions, which aim to reduce triceps surae (TS) impairment in patients with stroke, a scoping review was the study design that best fitted our needs. Scoping reviews are employed to map available literature on a novel, wide-ranging topic, to examine how research is conducted, highlighting any present gaps, and steering the scientific community towards filling these gaps [15].

In this study, a scoping review was performed to analyze current available evidence pertaining to PT interventions used with adult patients with stroke and relieve at least one of the causes underlying EFD. The focus was kept on the evaluation methods used by the authors to separately assess the various components that could be the underlying cause of EFD and on the methodological procedures used for data analysis. In this scoping review, clinicians and researchers can find an easy-to-consult summary on the methods used to assess PT interventions on EFD. Suggestions to improve everyday practice when dealing with clinical data have been provided to support further studies.

## 2 Methods

The methodology used for this scoping review is described by Tricco et al. [16] and is an extension of PRISMA statement for systematic reviews [17]. It consists of five stages, as described below.

### 2.1 Stage 1: Identifying the research question

The leading question for this investigation was: Which are the evaluation methods employed in literature to assess the effectiveness of PT interventions in patients with stroke with TS spasticity and EFD?

### 2.2 Stage 2: Identifying relevant studies

Comprehensive and systematic searches were developed by two researchers and a scientific librarian. The research was conducted in May 2021 with the following databases: Medline, Cinahl, and Cochrane. No time limitations were set and only articles published in English or Italian were considered eligible.

Keywords searched included “equinus deformity”, “stroke”, “physiotherapy”, “rehabilitation”, “extracorporeal shock waves”, “dry needling”, “stretching”, “ultrasound”, “vibration”, “tens”, “electric stimulation”, “muscle spasticity”, “spastic paresis”. When available, Medical Subjects Headings (MeSH) were included to ensure consistency of search terms. The complete search strategies can be consulted in Table 1. Additional papers were included by hand searching, retrieving them from bibliography of other studies.

**Table 1:** Search strategies of the investigated databases.

Database	Strategy	Notes
**Medline (PubMed)**	(triceps surae OR triceps OR gastrocnem* OR soleus OR "Equinus Deformity"[Mesh] OR equinus foot OR equinus deformity OR equinovarus foot OR equinovarus deformity) AND ("Stroke"[Mesh] OR Acute Stroke* OR Acute Cerebrovascular Accident* OR subacute Stroke OR "cerebrovascular disorders" [MeSH] OR "brain ischemia" [MeSH] OR "intracranial hemorrhages" [MeSH] OR "brain infarction" [MeSH] OR poststroke OR post-stroke OR cerebrovasc* OR cerebral* OR ischemi* OR haemorr*) AND (rehabilitation OR physiotherapy OR stretching OR shock-wav* OR "High-Energy Shock Waves"[Mesh] OR "Extracorporeal Shockwave Therapy"[Mesh] OR dry needling OR "Dry Needling"[Mesh] OR "Muscle Stretching Exercises"[Mesh] OR passive mobilization OR "Ultrasonic Waves"[Mesh] OR ultrasound* OR vibration therapy OR vibration* OR "Transcutaneous Electric Nerve Stimulation"[Mesh] OR tens OR "Electric Stimulation Therapy"[Mesh] OR electric stimulation) AND ("Muscle Spasticity"[Mesh] OR "Muscle Hypertonia"[Mesh] OR muscle spasticity OR muscle hypertonia OR spasticity OR spastic paresis OR overactivity OR spastic myopathy)	Search filters: • Adults: 19+ years
**Cochrane Database**	(triceps surae OR triceps OR gastrocnem* OR soleus OR equinus foot OR equinus deformity OR equinovarus foot OR equinovarus deformity) in Title Abstract Keyword AND (Stroke OR Acute Stroke* OR Acute Cerebrovascular Accident* OR subacute Stroke OR intracranial hemorrhages OR brain infarction OR poststroke OR post-stroke OR cerebrovasc* OR cerebral* OR ischemi* OR haemorr*) in Title Abstract Keyword AND (rehabilitation OR physiotherapy OR stretching OR shock-wav* OR dry needling OR passive mobilization OR ultrasound* OR vibration therapy OR vibration* OR tens OR electric stimulation) in Title Abstract Keyword AND (muscle spasticity OR muscle hypertonia OR spasticity OR spastic paresis OR overactivity OR spastic myopathy) in Title Abstract Keyword	
**Cinahl Database**	(MH "Equinus Deformity" OR triceps surae OR triceps OR gastrocnem* OR soleus OR equinus foot OR equinus deformity OR equinovarus foot OR equinovarus deformity) AND (MH "Cerebral Infarction" OR MH "Stroke" OR MH "Cerebrovascular Disorders" OR MH "Intracranial Hemorrhage" OR MH "Hypoxia-Ischemia, Brain" OR Acute Stroke* OR Acute Cerebrovascular Accident* OR subacute Stroke OR poststroke OR post-stroke OR cerebrovasc* OR cerebral* OR ischemi* OR haemorr* OR stroke) AND (Rehabilitation OR physiotherapy OR stretching OR shock-wav* OR dry needling OR passive mobilization OR ultrasound* OR vibration therapy OR vibration* OR tens OR electric stimulation or MH "Electric Stimulation" OR MH "Transcutaneous Electric Nerve Stimulation" OR MH "Stretching" OR MH "Dry Needling") AND (muscle spasticity OR muscle hypertonia OR spasticity OR spastic paresis OR overactivity OR spastic myopathy OR MH "Muscle Hypertonia" OR MH "Muscle Spasticity")	

### 2.3 Stage 3: Selecting studies

The eligibility criteria were set according to the PICO framework [18], as reported in Table 2.

**Table 2:** Eligibility criteria according to the PICO framework.

Element of PICO framework	Inclusion criteria	Exclusion criteria
Type of studies	• Primary studies Experimental, observational studies	• Secondary studies Reviews, meta-analyses • Full-text not available, even after contacting the authors • Conference abstracts
Population	• Adult patients (≥18 years) with stroke, with TS spasticity and EFD or EVFD	• Other neurological diseases • Cerebral palsy, multiple sclerosis, traumatic brain injury, spinal cord injury, Parkinson’s disease, etc. • Patients with drop foot due to dorsiflexors paresis without increased TS muscle tone
Intervention	• PT interventions performed without powered devices to reduce EFD • Stretching, dry-needling, cryotherapy, active PT • PT interventions performed with powered devices to reduce EFD • Shock waves, electrostimulation, TENS, vibrations, ultrasound	• Surgical treatments • Muscle-tendon lengthening, tendon transfers, neurotomies, etc. • Pharmacological treatments • Focal muscle inhibition (by botulinum-toxin, alcohol or phenol injections), global diffusion (by intrathecal baclofen) • Alternative therapy • Herbs, homeopathy, uniforms and suits
Comparison	• Any type of control treatment • No intervention, sham therapy, alternative therapy, usual care	/
Outcome	• At least one outcome measure assessing stroke-related TS impairment • Clinical or instrumental measures, passive manual manoeuvres, imaging	• Papers reporting only functional outcomes, without any measurement directly linked to TS impairment or structural changes 6-Minute-Walking test, Timed-Up&Go, etc.

TS *Triceps Surae;* EFD *Equinus Foot Deformity;* EVFD *Equino-varus Foot Deformity;* PT *Physiotherapy*.

### 2.4 Stage 4: Charting the data

Relevant titles and abstracts were screened according to the inclusion and exclusion criteria and full text papers were independently evaluated by two reviewers (IC and MCBO, two licensed PTs). When a consensus was not reached, a third researcher (AM) resolved any discrepancy.

### 2.5 Stage 5: Summarizing and reporting the data

Relevant data were extracted from papers and collected in a pre-defined Excel form (including first author, year of publication, intervention type, outcome measure, etc.) [19,20]. The form was updated once during the data extraction process to maximize the accuracy of this study, in line with the methodology for scoping reviews [20]. Data were finally collected in tables and classified by author and year of publication, intervention types and clinical and instrumental outcome measures. Findings were presented in a narrative synthesis, grouped by type of intervention.

## 3 Results

**Fig 1:**
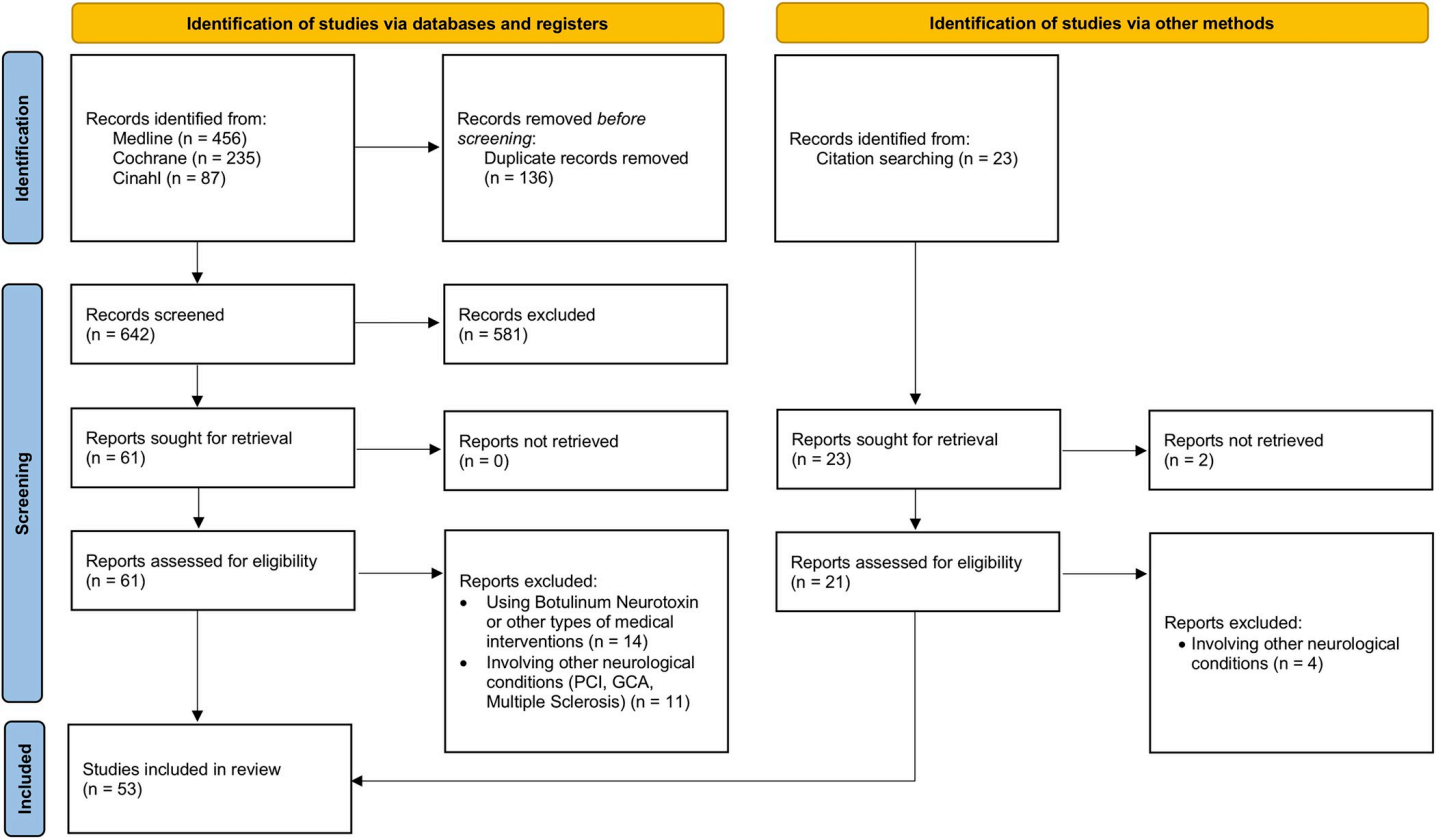
Flow Chart of the literature search on assessment modalities of physiotherapy interventions for equino-varus foot deformity with triceps spasticity in patients with stroke.

The search led to the identification of 778 articles and 642 papers remained after removing all duplicates. Of these, 61 papers were selected for full-text screening based on the title and abstract and 36 were included in the scoping review. In addition, 17 studies were identified by hand searching and were later included, for a total of 53 studies. The flow chart in Fig 1 follows the PRISMA guidelines [17].

The selected papers were published between 2001 and 2020, with samples ranging from 1 to 83 adult patients with EFD and TS spasticity following stroke. Thirty-two of the included studies were randomized controlled trials (RCTs), while the remaining had study designs without randomization or controls.

The rationale followed by authors in delivering one treatment over another was that each author could intervene on different factors causing EFD (e.g., either active, reflex or passive). Details of the components addressed by the included studies as stated by authors are presented in Table 3.

**Table 3:** Targeted components of the equino-varus foot deformity addressed by the physiotherapeutic treatments according to the authors of the included studies.

Intervention	Generic expected effects	Specific expected effects			
		Active components	Passive components of connective tissues	Reflex components	Muscle properties
**Stretching**	• Spasticity [21–26] • Range of motion [21,22,25–30]		• Stiffness [21,23–25,27–31]	• H-reflex latency and H/M ratio [22–25,27,31]	• Sarcomere number [21,27] • Architectural parameters [22]
**Shock waves**	• Spasticity [32–40] • Range of motion [32–37,39]		• Stiffness and fibrosis [32,38]	• Hypertonia [38] • Electrophysiological parameters [34,40]	• Architectural parameters [39]
**Electrostimulation**	• Spasticity [41–47] • Range of motion [41,42,45,46]	• Muscle strength [41–43,45]	• Stiffness [46]	• H-reflex [46] • Inhibitory mechanisms [42,45–47]	
**Dry needling**	• Spasticity [48–53] • Range of motion [48,53]		• Stiffness [52] • Cytoskeletal structures [52] • Endplate zones [48,51,52]	• H-reflex [53] • Antinociceptive reflex [52]	• Blood flow [48,51] • Architectural parameters [49]
**TENS**	• Spasticity [54–59] • Range of motion [55]	• Muscle strength [58,59]		• H-reflex [55,56,58,59] • Inhibitory mechanisms [54,56,57]	
**Whole-body vibrations**	• Spasticity [60–65] • Range of motion [60,62]	• Muscle strength [62,63,65]	• Stiffness [61]	• Inhibitory mechanisms [61–65] • Electrophysiological parameters [60,61]	• Blood perfusion and tissue oxygenation [61]
**Ultrasound**	• Spasticity [33,55,66–68] • Range of motion [33,55,66–68]		• Stiffness [55]	• Stretching sensitivity [55]	
**Cryotherapy**	• Spasticity [57,69,70]			• H-reflex [57,69,70]	• Blood flow and local metabolism [69,70]
**Physiotherapist-guided physical exercise**	• Spasticity [71,72]	• Muscle strength [71,72]		• Cocontraction [71]	

### 3.1 Stretching

Twelve studies used stretching maneuvers. Two of them were RCTs. Their characteristics can be seen in Table 4.

**Table 4:** Assessment methods of studies using stretching for the treatment of triceps spasticity after stroke.

Author and year	Study design	Intervention dosage	Control group	Clinical outcomes	Instrumental outcomes
Pradines et al. 2019 [21]	RCT	Usual care three times a week + 15-minute stretching Once a day, for 1 year (self-managed)	Usual care three times a week, for 1 year	**Tardieu Angle** 10maS	Fascicle Length, Muscle Thickness
Ghasemi et al. 2018 [22]	RCT	3-minute functional stretching Three times a week, for one month	Usual care	**MMAS** (mean, median) presence of clonus, pROM	Achilles tendon reflex excitability, H-reflex latency, H/M ratio, Pennation Angle, Muscle Thickness, Fascicle Length
Pradines et al. 2018 [23]	Single-arm trial	15-minute stretching Once a day, for 1 year (self-managed)	n.a.	**Tardieu Angle** Csh, 10maS	n.a.
Gao et al. 2011 [27]	Non-randomized trial	60-minute stretching (12 repetition lasting 5 min each) with intelligent device One single session	10 healthy subjects		**Torque-angle ratio** (stiffness, strength) Achilles tendon length
Bakheit et al. 2005 [24]	Non-randomized trial	IG1: 20-minute isotonic stretching (with weight bearing) IG2: 20-minute stretching (without weight bearing) IG3: 20-minute isokinetic stretching One single session with intelligent device	21 healthy subjects		**H/M ratio**, H-reflex latency
Chung et al. 2005 [25]	Non-randomized trial	30-minute stretching with intelligent device One single session	10 healthy subjects	pROM	**Peak reflex torque, ATR threshold**, Torque-angle ratio (stiffness), Maximum Voluntary Contraction
Maynard et al. 2005 [31]	Non-randomized trial	IG1: 20-minute isotonic stretching (with weight bearing) IG2: 20-minute stretching (without weight bearing) IG3: 20-minute isokinetic stretching One single session with intelligent device	21 healthy subjects		**Kinematic, dynamic and spatio-temporal parameters** through gait analysis
Selles et al. 2005 [28]	Single-arm trial	45-minute stretching with intelligent device 3 times a week, for one month	n.a.	pROM, aROM, 10maS, VASs	**Torque-angle ratio** (stiffness, viscosity), MVC (strength), ATR excitability
Yeh et al. 2005 [26]	Non-randomized trial	IG1: 30-minute constant-angle stretching with intelligent device One single session	IG2: 30-minute constant-torque stretching with intelligent device	**MAS** (range) pROM	Reactive Torque
Bressel et al. 2002 [29]	Non-randomized trial	IG1: 30-minute static stretching with intelligent device One single session	IG2: 30-minute cycling stretching, one single session 1 week after	10MWT	**Ankle Joint angle and passive torque** (stiffness), EMG activity
Zhang et al. 2002 [30]	Single-arm trial	Stretching with intelligent device One single session	n.a.	aROM, pROM	**Torque-angle ratio** (stiffness, viscosity), Achilles tendon reflex excitability
Tsai et al. 2001 [73]	Single-arm trial	30-minute stretching on a tilt-table One single session	n.a.	**MAS** (mean) pROM	H/M ratio, F/M ratio

RCT *Randomized Controlled Trial*, IG *Intervention Group*, CG *Control Group*, n.a. *not available*, RCT *Randomized Controlled Trial*, TS *Tardieu Scale*, 10maS *10 meter ambulation Scale*, FL *Fascicle Length*, MT *Muscle Thickness*, MMAS *Modified Modified Ashworth Scale*, pROM *passive Range of Motion*, ATR *Achilles Tendon Reflex*, PA *Pennation Angle*, Csh *Coefficient of Shortening*, MAS *Modified Ashworth Scale*, aRoM *active Range of Motion*, MVC *Maximal Voluntary Contraction*, VASs *Visual Analogue Scale score*, RT *Reactive Torque*, EMG *Electromyography*, 10MWT *10-m Walking Test*.

The effect of stretching on EVD was assessed both by clinical and instrumental measures. Six studies measured passive range of motion (ROM) [22,25,26,28,30,73]. The Modified Ashworth Scale (MAS) was employed in three of them [22,26,73], while Pradines and colleagues used the Tardieu Angle [21,23]. The remaining seven papers used instrumental measures as the primary outcome for the quantification of spasticity or stiffness, such as the H/M ratio (i.e., the ratio between the maximum amplitude of H-wave and M-wave) [24], the Achilles Tendon Reflex Excitability [22,25], kinematic data extracted by gait analysis [31] or the torque-angle ratio [27–30].

### 3.2 Shock waves

The effect of shock waves on TS spasticity was the main topic in nine studies. Study characteristics can be viewed in Table 5. Four were RCTs. Eight out of nine studies used MAS or its subsequent versions, [32–37,39,40]. Wu and colleagues assessed TS spasticity using the Tardieu Angle [36]. Other frequently used clinical measurements were active and passive ankle ROM and gait velocity retrieved from different functional tests. Only Sawan’s group employed an instrumental outcome measure—the H/M ratio—as the primary outcome for spasticity quantification [38]. Almost all studies used instrumental evaluations as secondary outcomes.

**Table 5:** Assessment methods employed by studies using shock waves for the treatment of triceps spasticity after stroke.

Author and year	Study design	Intervention dosage	Control group or other intervention group	Clinical outcomes	Instrumental outcomes
Lee et al. 2019 [32]	RCT	ESWT (2000 shots, 0.1 mJ/mm2) in the medial gastrocnemius + physiotherapy One single session	Sham stimulation + physiotherapy	**MAS** (mean) pROM, FMA	Achilles Tendon Length, Fascicle Length, Muscle Thickness, Pennation Angle
Radinmehr et al. 2019 [33]	RCT	IG1: rSWT (2000 shots, 0.34 mJ/mm2) in the gastrocnemius One single session	IG2: Ultrasound therapy	**MMAS** (median) aROM, pROM, TUG	H-reflex latency, PPFT
Wu et al. 2018 [36]	RCT	IG1: rSWT (1500 shots, 0.1 mJ/mm2) in the gastrocnemius and in the soleus Once a week, for 3 weeks	IG2: fSWT (1500 shots, 2.0 bar) in the gastrocnemius and in the soleus	**MAS, Tardieu Angle** (mean and median) pROM, 10MWT	Dynamic Foot Contact Area
Sawan et al. 2017 [38]	Non-randomized trial	ESWT (1500 shots) in the gastrocnemius + physiotherapy Once a week, for 6 weeks	Sham stimulation + physiotherapy	aROM, 10MWT	**H/M ratio**
Taheri et al. 2017 [35]	RCT	fSWT (1500 shots, 0.1 mJ/mm2) in the gastrocnemius + stretching exercises Once a week, for 3 weeks	Stretching exercises	**MAS** (mean) VAS, pROM, CS, 3MWT, LEFS	n.a.
Radinmehr et al. 2017 [34]	Single-arm trial	rSWT (2000 shots, 0.34 mJ/mm2) in the gastrocnemius One single session	n.a.	**MMAS** (median) aROM, pROM, TUG	H-reflex, H/M ratio, PPFT
Santamato et al 2014 [39]	Single-arm trial	fSWT (1500 shots, 0.1 mJ/mm2) in the gastrocnemius and in the soleus One single session	n.a.	**MAS** (mean) pROM	Tibial Nerve Conduction, F-wave latency
Moon et al. 2013 [37]	Single-arm trial	One sham fSWT session physiotherapy + fSWT (1500 shots, 0.089 mJ/mm2) in the gastrocnemius Once a week, for 3 weeks	n.a.	**MAS** (mean) CS, pROM, FMA	PET, TTAs
Sohn et al. 2011 [40]	Non-randomized trial	fSWT (1500 shots, 0.1 mJ/mm2) in the gastrocnemius One single session	10 healthy subjects (same treatment)	**MAS** (mean)	F-waves latency, H-reflex latency, H/M ratio, tibial nerve conduction

RCT *Randomized Controlled Trial*, IG *Intervention Group*, *CG Control Group*, n.a. *not available*, RCT *Randomized Controlled Trial*, MAS *Modified Ashworth Scale*, ESWT *External Shock-Wave Therapy*, aROM *active Range of Motion*, FMA *Fugl-Meyer Assessment*, ATL *Achilles Tendon Length*, FL *Fascicle Length*, MT *Muscle Thickness*, PA *Pennation Angle*, rSWT *radial Shock-Wave Therapy*, MMAS *Modified Modified Ashworth Scale*, pROM *passive Range of Motion*, TUG *Timed Up and Go test*, PPFT *Passive Plantarflexor Torque*, fSWT *focused Shock-Wave Therapy*, TS *Tardieu Scale*, 10MWT *10-m Walking Test*, DFCA *Dynamic Foot Contact Area*, VAS *Visual Analogue Scale*, CS *Clonus Score*, 3MWT *3-m Walking Test*, LEFS *Lower Extremity Functional Score*, TNC *Tibial Nerve Conduction*, PET *Peak Eccentric Torque*, TTAs *Torque Threshold Angles*

### 3.3 Electrical stimulation

Seven studies employed electrical stimulation to treat spasticity and lack of strength in patients with stroke with EFD. Six were RCTs. Study characteristics are presented in Table 6.

**Table 6:** Assessment methods of studies using electrical stimulation for the treatment of triceps spasticity after stroke.

Author and year	Study design	Intervention dosage	Control group or other intervention group	Clinical outcomes	Instrumental outcomes
Ganesh et al. 2018 [41]	RCT	IG1: 80-min rehabilitation (with task-oriented exercises) + 10-minute Faradic Currents (100 Hz) on peroneal and tibial nerve IG2: 80-min rehabilitation (with task-oriented exercises) + 10-minute Russian Current (2500 Hz) on peroneal and tibial nerve Five days a week, for 6 weeks	80-min rehabilitation (with task-oriented exercises)	**MMAS** (range) aROM, pROM, mEFAP	n.a.
Yang et al. 2018 [42]	RCT	IG1: 20-minute NMES (50 Hz) on tibialis anterior + 15-min gait training IG2: 20-minute NMES (50 Hz) on medial gastrocnemius + 15-min gait training Three days a week, for 7 weeks	20-min stretching and ROM exercises + 15-min gait training	**MAS** (mean) Muscle strength	Gastrocnemius dynamic EMG-activity, aROM (electronic goniometer during gait), Gait analysis parameters
Sharif et al. 2017 [43]	RCT	30-minute FES (40 Hz) on tibialis anterior + usual rehabilitation program Five days a week, for 6 weeks	10-min electrical stimulation on tibialis anterior + usual rehabilitation program	**MAS** (mean) Fugl-Meyer Assessment, Berg Balance Scale, TUG, Gait Dynamic Index	
Suh et al. 2014 [44]	RCT	30-min Bobath approach + 60-minute Interferential Currents (100 Hz) on the gastrocnemius One single session	30-min Bobath approach + sham Interferential Currents	**MAS** (mean) Functional Reach Test, Berg Balance Scale, TUG, 10-maS	n.a.
Sabut et al. 2011 [45]	Non-randomized trial	1-h rehabilitation program + 30-min electrical stimulation (35 Hz) on tibialis anterior Five days a week, for 3 months	1-h rehabilitation program, five days a week, for 3 months	**MAS** (mean) MRC, aROM, pROM, FMA	n.a.
Bakhtiary et al. 2008 [46]	RCT	10-min infrared + 15-min Bobath approach + 9-minute Faradic Currents (100 Hz) on the tibialis anterior 20 daily sessions	10-min infrared + 15-min Bobath approach	**MAS** (mean) pROM, MRC	H/M ratio
Chen et al. 2005 [47]	RCT	20-minute electrical stimulation (20 Hz) on the gastrocnemius Six days a week, for 1 month	Sham electrical stimulation	**MAS** (count) 10-maS	F/M ratio, H-reflex latency

RCT *Randomized Controlled Trial*, IG *Intervention group*, CG *Control Group*, n.a. *not available*, RCT *Randomized Controlled Trial*, MCSS *Modified Composite Spasticity Score*, MMAS *Modified Modified Ashworth Scale*, aROM *active Range of Motion*, pROM *passive Range of Motion*, mEFAP *modified Emory Functional Ambulation Profile*, NMES *Neuromuscular Electrical Stimulation*, MAS *Modified Ashworth Scale*, EMG *Electromyography*, FES *Functional Electrostimulation*, TUG *Timed Up and Go*, FMA *Fugl-Meyer Assessment*, BBS *Berg Balance Scale*, GDI *Gait Dynamic Index*, 10MWT *10-m Walking Test*, FRT *Functional Reach Test*, MRC *Medical Research Council*, 10-maS *10-m ambulation Speed*.

To assess spasticity all trials used MAS or its subsequent versions [41–47]. Other clinical outcome measures were ROM, muscle strength, gait performance scores (Timed Up and Go, modified Emory Functional Ambulation Profile, 10-Metre Ambulation Speed), and stability scores (Berg Balance Scale, Fugl-Meyer Assessment, Gait Dynamic Index).

Three studies also included instrumental measures. Yang and Colleagues used dynamic electromyography during gait analysis to measure TS activity during walking [42]. The remaining two studies measured the H-reflex and other neurophysiological parameters [46,47].

### 3.4 Dry needling

Six studies performed dry needling to treat either the passive or active components of EFD in patients with stroke. Four were RCTs. Their characteristics can be seen in Table 7.

**Table 7:** Assessment methods of studies using dry needling for the treatment of triceps spasticity after stroke.

Author and year	Study design	Intervention dosage	Control group	Clinical outcomes	Instrumental outcomes
Ghannadi et al. 2020 [48]	RCT	Dry needling with fast-in and fast-out technique in the gastrocnemius 3 sessions in a week	Sham dry needling with blunted needle	**MMAS** (mean, median) TUG, Single Leg Stance, 10MWT, Barthel Index, aROM, pROM	Pennation Angle, Muscle Thickness
Hadi et al. 2018 [49]	Single-arm trial	Dry needling in the gastrocnemii and in the soleus One single session	n.a.	**MMAS** (median) TUG	Pennation Angle, Muscle Thickness, Fascicle Length
Sànchez-Mila et al. 2018 [50]	RCT	Dry needling with fast-in and fast-out technique in the tibialis posterior + Bobath approach One single session	Bobath approach	**MMAS** (median) FMA	Limits of Stability test (dynamic posturography)
Calvo et al. 2016 [51]	Single-arm trial	Dry needling in the gastrocnemii One single session	n.a.		**Tensiomyography parameters**
Salom-Moreno et al. 2014 [52]	RCT	Dry needling with fast-in and fast-out techniques in the gastrocnemius and tibialis anterior One single session	No intervention	**MMAS** (median)	PPT, Baropodometric values (static)
Fink et al. 2004 [53]	RCT	Dry needling in acupuncture points Two times a week, for 1 month	Sham needles placed in non-acupoints	**MAS** (mean) aROM, pROM, 2MWT	H-reflex, H/M ratio

RCT *Randomized Controlled Trial*, IG *Intervention Group*, CG *Control Group*, n.a. *not available*, RCT *Randomized Controlled Trial*, MMAS *Modified Modified Ashworth Scale*, TUG *Timed Up and Go*, 10MWT *10-m Walking Test*, aROM *active Range of Motion*, pROM *passive Range of Motion*, PA *Pennation Angle*, MT *Muscle Thickness*, SLS *Single Leg Stance*, BI *Barthel Index*, FL *Fascicle Length*, FMA *Fugl-Meyer Assessment*, LoS test *Limits of Stability test*, MD *Maximal Displacement*, PPT *Pressure Pain Thresholds*, 2MWT *2-m Walking Test*.

MAS score was the main clinical outcome measure for assessing the effects of the intervention in five out of six papers [48–50,52,53], while Calvo and colleagues used an instrumental measure called Maximal Radial Muscle Displacement which is computed by tensiomyography [51]. Secondary clinical measures included functional performance scales (Timed Up and Go, 10-Meter Walking Test, Fugl-Meyer Assessment, Single Leg Stance). Other instrumental outcomes used by authors were associated to the structural characteristics of muscles (pennation angle, muscle thickness, fascicle length) and to the patients’ static and dynamic stability.

### 3.5 Transcutaneous electrical nerve stimulation (TENS)

Six studies used TENS to treat spasticity in patients with stroke with EFD. Five were RCTs. Their characteristics can be seen in Table 8.

**Table 8:** Assessment methods of studies using Transcutaneous Electrical Nerve Stimulation (TENS) for the treatment of triceps spasticity after stroke.

Author and year	Study design	Intervention dosage	Control group	Clinical outcomes	Instrumental outcomes
Koyama et al. 2016 [54]	Single-arm trial	30-min TENS (50/100/200 Hz) in the peroneal nerve (0.25 ms, 40s ON/10s OFF) One single session	n.a.	n.a.	**Reciprocal Inhibition, Presynaptic Inhibition** H-reflex latency
Picelli et al. 2014 [55]	RCT	IG1: 15-minute TENS (100 Hz, 0.3 ms) on the tibial nerve Five days a week, for 2 weeks	IG2: 10-minute continuous ultrasound in the triceps, five days a week for 2 weeks IG3: BoNT-A injections in the gastrocnemius	**MAS** (median) pROM	n.a.
Cho et al. 2013[56]	RCT	30-min Bobath rehabilitation + 60-min TENS (100 Hz, 0.2 ms) on the gastrocnemius One single session	30-min Bobath rehabilitation + sham TENS	**MAS** (mean)	**Plantarflexors resistance** Static and dynamic postural sway length
Martins et al. 2012 [57]	RCT	IG1: 30-minute TENS (100 Hz, 0.06 ms) in the posterior tibialis nerve One single session	IG2: 30-minute cryotherapy (applied at the same group the following day) CG: 40 assessments of non-affected limbs before treatments	n.a.	**H/M ratio** H-reflex latency, Tibialis anterior EMG activity
Yan et al. 2009 [58]	RCT	60-min usual rehabilitation + 60-minute TENS (100 Hz, 0.2 ms) on the acupuncture points of the lower limb Five days a week, for 3 weeks	CG1: 60-min usual rehabilitation + sham TENS CG2: 60-min usual rehabilitation only	**CSS** (median) TUG	MIVC of tibialis anterior and gastrocnemius, Co-contraction of plantarflexors
Ng et al. 2007 [59]	RCT	IG1: 60-minute TENS (100 Hz, 0.2 ms) IG2: 60-minute TENS (100 Hz, 0.2 ms) + 60-minute task-related training Five days a week, for 1 month	CG1: Sham TENS + 60-minute task-related training CG2: No intervention	**CSS** (mean)	MIVC of dorsiflexors and plantarflexors, Gait velocity (instrumented carpet)

RCT *Randomized Controlled Trial*, IG *Intervention Group*, CG *Control Group*, n.a. *not available*, RCT *Randomized Controlled Trial*, TENS *Transcutaneous Electrical Stimulation*, PI *Presynaptic Inhibition*, RI *Reciprocal Inhibition*, MAS *Modified Ashworth Scale*, BoNT-A *Botulinum Neurotoxin Type-A*, pROM *passive Range of Motion*, HHD *Handheld Dynamometer*, EMG *Electromyography*, CSS *Composite Spasticity Scale*, MIVC *Maximum Isometric Voluntary Contraction*, TUG *Timed Up and Go*.

Four papers performed a clinical evaluation. Two used MAS [55,56], while Yan and Ng, employed another clinical scale called the Composite Spasticity Scale (CSS) [58,59].

The remaining two papers used instrumental neurophysiological measures: H/M ratio, H-reflex latency and the amount of both reciprocal and presynaptic inhibition [54,57]. Moreover, Ng and Cho measured gait velocity and postural stability in dynamic conditions with the aid of instrumented devices [56,59].

### 3.6 Whole-body vibrations

In six studies, patients with EFD were given whole-body vibration therapy to treat spasticity. Four were RCTs. Their characteristics can be seen in Table 9.

**Table 9:** Assessment methods of studies using vibration for the treatment of triceps spasticity after stroke.

Author and year	Study design	Intervention dosage	Control group	Clinical outcomes	Instrumental outcomes
Huang et al. 2020 [61]	RCT	Vertical WBV (30 Hz, 1.5 mm amplitude), bent knee standing on a platform Five 1-min bouts, one single session	Sham WBV on the same platform, 2 days after	n.a.	**H/M ratio** (standing) Stiffness, Intramuscular blood perfusion (color power Doppler)
Miyara et al. 2020 [62]	Non-randomized trial	5-minute tri-planar WBV (30Hz, 4–8 mm amplitude), seated + simultaneous triceps stretching One single session	Healthy subjects	**MAS** (median) aROM, pROM	n.a.
Alp et al. 2018 [63]	RCT	Stretching + exercises + 5-minute vertical WBV (40 Hz, 4 mm amplitude), bent knee standing on a platform Three days a week, for 1 month	Stretching + exercises + sham WBV on the same platform	**MAS** (median) FIM, 10maS	n.a.
Miyara et al. 2018 [62]	Single-arm trial	5-minute tri-planar WBV (30Hz, 4 mm amplitude), seated One single session	n.a.	**MAS** (median) aROM, pROM	F-waves, F/M ratio
Pang et al. 2013 [64]	RCT	Vertical WBV (20–30 Hz, 0.44–0.6 mm amplitude), standing on a platform while performing specific exercises Three days a week, for 2 months	Sham WBV on the same platform while performing specific exercises	**MAS** (median) Chedoke McMaster Stroke Assessment	Muscle power (dynamometer)
Chan et al. 2012 [65]	RCT	Vertical WBV (12 Hz, 4 mm amplitude), bent knee standing on a platform Two 10-min bouts, one single session	Sham WBV on the same platform	**MAS** (mean) VAS, TUG, 10maS	ATR excitability, H-reflex, H/M ratio, Static Foot Contact Area

RCT *Randomized Controlled Trial*, IG *Intervention Group*, CG *Control Group*, n.a. *not available*, RCT *Randomized Controlled Trial*, MAS *Modified Ashworth Scale*, WBV *Whole Body Vibration*, IBP *Intramuscular Blood Perfusion*, FIM *Functional Independence Measurement*, 10maS *10-m ambulation Speed*, aROM *active Range of Motion*, pROM *passive Range of Motion*, CMSA *Chedoke McMaster Stroke Assessment*, VAS *Visual Analogue Scale*, TUG *Timed Up and Go*, SFCA *Static Foot Contact Area*, ATR *Achilles Tendon Reflex*.

Five out of six studies selected MAS clinical assessment [60,62–65]; other clinical measures employed were gait velocity, stability, and joint ROM.

One study by Huang and colleagues assessed patients using the H/M ratio while standing [61], as primary outcome. Three other authors added instrumental evaluations assessing either the neurophysiological parameters of the reflex arc function (the tendon reflex excitability, the F-waves, or the F/M ratio), or the biomechanical variables (weight distribution under the foot, or muscle power).

### 3.7 Ultrasound

Five studies employed ultrasound to treat spasticity in patients with EFD. All were RCTs. Their characteristics can be seen in Table 10.

**Table 10:** Assessment methods of studies using ultrasound for the treatment of triceps spasticity after stroke.

Author and year	Study design	Intervention dosage	Control group	Clinical outcomes	Instrumental outcomes
Radinmehr et al. 2019 [33]	RCT	IG1: 10-minute continuous ultrasound (1MHz, 1.5 W/cm2) in the gastrocnemius One single session	IG2: Radial shock-wave therapy	**MMAS** (median) aROM, pROM, TUG	H-reflex latency, PPFT
Picelli et al. 2014 [55]	RCT	IG1: 10-minute continuous ultrasound (1 MHz, 1.5 W/cm2) in the triceps Five days a week, for 2 weeks	IG2: 15-minute TENS (100 Hz, 0.3 ms) on the tibial nerve, five days a week for 2 weeks IG3: BoNT-A injections in the gastrocnemius	**MAS** (median) pROM	n.a.
Sahin et al. 2011 [66]	RCT	Stretching + 10-minute continuous ultrasound (1.5 W/cm2) Five days a week, for 1 month	Stretching five days a week, for 1 month	**MAS** (mean) aROM, pROM, BMRS, FIM	H/M ratio
Ansari et al. 2009 [68]	RCT	IG1: 10-minute continuous ultrasound (1 MHz, 1.5 W/cm2) in the triceps One single session	IG2: 20-minute infrared (500W), one single session	**MAS** (median) aROM, pROM	H/M ratio
Ansari et al. 2007 [67]	RCT	10-minute continuous ultrasound (1 MHz, 1.5 W/cm2) in the triceps Three days a week, for 5 weeks	Sham ultrasound in the triceps, three days a week for 5 weeks	**MAS** (mean, range) aROM, pROM	H/M ratio

RCT *Randomized Controlled Trial*, IG *Intervention Group*, CG *Control Group*, n.a. *not available*, RCT *Randomized Controlled Trial*, MMAS *Modified Modified Ashworth Scale*, aROM *active Range of Motion*, pROM *passive Range of Motion*, TUG *Timed Up and Go*, PPFT *Passive Plantarflexor torque*, MAS *Modified Ashworth Scale*, BoNT-A *Botulinum Neurotoxin Type-A*, BMRS *Brunnstrom Motor Recovery Stage*, FIM *Functional Independence Measurement*.

All authors used MAS as the main outcome [33,55,66–68]. Passive ROM was also assessed by all authors, along with several other functional tests. The most frequent instrumental measurement used were H/M ratio or H-reflex latency.

### 3.8 Cryotherapy

Three studies used cryotherapy. All were RCTs. Their characteristics can be seen in Table 11.

**Table 11:** Assessment methods of studies using cryotherapy for the treatment of triceps spasticity after stroke.

Author and year	Study design	Intervention dosage	Control group or other intervention group	Clinical outcomes	Instrumental outcomes
Alcantara et al. 2019 [69]	RCT	20-min cryotherapy (ice pack of 1 kg) One single session	1-kg pack filled with sand at room temperature	**MAS** (median)	Dorsiflexors and plantarflexors strength (Isokinetic dynamometer), Gait analysis parameters
Garcia et al. 2019 [70]	RCT	20-min cryotherapy (ice pack of 1 kg) One single session	1-kg pack filled with sand at room temperature	**MAS** (mean, count) Joint position sense	n.a.
Martins et al. 2012 [57]	RCT	IG1: 30-minute cryotherapy (applied at the same group the second day) One single session	IG2: 30-minute TENS (100 Hz, 0.06 ms) in the posterior tibialis nerve CG: 40 assessments of non-affected limbs before treatments		**H/M ratio** H-reflex latency, Tibialis anterior EMG activity

RCT *Randomized Controlled Trial*, IG *Intervention group*, CG *Control Group*, n.a. *not available*, RCT *Randomized Controlled Trial*, MAS *Modified Ashworth Scale*, pROM *passive Range of Motion*, 6MWT *6-m Walking Test*.

Authors Alcantara and Garcia, chose MAS score as the primary clinical outcome in their studies [69,70]. In their first paper they also investigated the joint position sense of patients (i.e., how patients perceived their joint positions) [70].

When considering instrumental outcomes, Martins and Colleagues set the H/M ratio as their primary measurement, together with H-reflex latency and electromyographic activity of the tibialis anterior during maximum contraction [57]. Alcantara measured muscle strength with an isokinetic dynamometer and performed a gait analysis collecting many parameters, including ankle joint angles [69].

### 3.9 Physiotherapist-guided physical exercise

Two studies provided physical exercise programs led by physiotherapists. Both were RCTs. Their characteristics can be seen in Table 12.

**Table 12:** Assessment methods of studies providing physiotherapist-guided physical exercise interventions for the treatment of triceps spasticity after stroke.

Author and year	Study design	Intervention dosage	Control group or other intervention group	Clinical outcomes	Instrumental outcomes
Zhang et al. 2016 [71]	RCT	40-minute water-based exercises Five days a week, for 2 months	40-min land-based exercises	**MAS** (median) Functional Ambulation Category, Barthel Index	MVIC of dorsiflexors and plantarflexors
Akbari et al. 2006 [72]	RCT	Strengthening, balance, and functional exercises Three days a week, for 1 month	Balance and functional exercises	**MAS** (mean)	Muscle strength (dynamometer)

RCT *Randomized Controlled Trial*, IG *Intervention group*, CG *Control Group*, n.a. *not available*, RCT *Randomized Controlled* Trial, MAS *Modified Ashworth Scale*, MVIC *Maximum Voluntary Isometric Contraction*, FAC *Functional Ambulatory Category*, BI *Barthel Index*, EMG *Electromyography*.

Both studies assessed TS spasticity with MAS [71,72]. Zhang also performed two functional tests [71]. Again, both works measured muscle activity parameters with instrumental tools: one computed the Maximum Voluntary Isometric Contraction through surface electromyography [71], while the other measured strength with a dynamometer [72].

## 4 Discussion

### 4.1 An overview on the main findings

The main purpose of this scoping review was to collect and analyze all evaluation methods used in literature to assess the efficacy of PT interventions for the treatment of EFD due to TS spasticity in patients with stroke. In the manuscripts, all treatments performed by physiotherapists were analyzed. It is worth noting that laws that govern physical therapists’ ability to perform specific treatments (e.g., dry needling) may vary among countries.

Most of the included studies were RCTs, comparing PT interventions to placebo treatments or usual care (see Tables 4–12). Six studies [24,25,27,31,40,62] compared an intervention group with patients with stroke to a control group of healthy subjects. This is a limitation since it does not allow for an appropriate comparison between groups and the only viable analysis to be made is within-group difference only prior to, and after the intervention. The same applies for the eleven studies with a single-arm trial design [23,28,30,34,37,39,49,51,54,60,73]. Four further studies [26,29,38,45] did not randomize participant allocation. This choice could have been made because of clinical feasibility issues. However, this limits the considerations that can be drawn from the findings and, when possible, should be avoided.

#### 4.1.1 Clinical measures of spasticity

From this scoping review, it emerges that almost all authors stated that their main goal was to reduce TS spasticity (see Table 3). Most of them employed MAS to estimate it. MAS was in fact used in 37 studies out of 53 (69%) of the included studies. Regardless of the therapy administered, almost all authors chose MAS as the main outcome measurement, with the exception of stretching and TENS. MAS was both used as a primary outcome and as an inclusion criterion. In fact, almost all samples of the included studies had to score at least one point at MAS to be considered eligible. Since MAS measures an overall increase in tone, this could have led to the inclusion of patients with EFD only due to TS retraction, instead of TS spasticity [74–77]. Because EFD can have several underlying potential causes, these patients might not have been sensitive to the treatments provided, especially those focusing solely on the reflex components [78].

Twelve included studies used MAS, citing the works by Bohannon and Smith, who conducted a study in 1986, on a largely heterogeneous sample of neurological patients [79]. Their aim was to increase the inter-assessor reliability of the scale through the introduction of the 1+ score between 1 and 2 levels of the original Ashworth Scale [79]. Nine other papers cited Ghotbi and Ansari’s works, who later derived the Modified-MAS by removing once more the 1+ score [80,81]. Both references are quite outdated. We believe that most authors used those references out of habit, as confirmed by further five studies that assessed spasticity with MAS without even justifying its use. However, a careful analysis could highlight the methodological shortcomings of the studies that developed these assessment scales. In fact, since the 1990s, the scientific community began questioning the use of MAS as an appropriate scale for measuring spasticity, suggesting the presence of a cultural bias that sometimes seems to persist even today [74]. Despite providing satisfactory repeatability among assessors, thanks to the development of the modified versions [82], MAS still fails in terms of construct validity on larger muscles (e.g., TS muscle) [74,83], because of its complex outcome measurements that do not solely quantify spasticity, but an overall resistance to passive movement–otherwise known as muscle tone [84].

Given the limits of MAS, there is a need for alternative clinical scales. The Tardieu Scale and the Tardieu Angle have good construct validity [85,86]. These outcome measures try to isolate spasticity, according to its definition of a velocity-dependent response to phasic stretch [78,87]. The Tardieu Angle is used to identify a brake caused by passive resistance or by spasticity by performing two stretch maneuvers as slowly and as quickly as possible. The difference between the two angles is called Tardieu Angle and it measures how much spasticity contributes to ROM limitation [88]. Despite its good construct validity, only three studies, included in our review, used this method. It would be appropriate to increase its employment in future investigations. Li and colleagues analyzed the psychometric properties of MAS and Tardieu Scale in a cohort of patients with stroke and suggested using the latter during the clinical assessment of ankle plantarflexors due to better inter and intra-assessor reliability [89]. Another clinical scale used in two studies was CSS, which ordinally evaluates tendon jerks, resistance to passive stretch, and clonus [90].

The measurement of passive ROM was employed in 24 studies, crosswise for almost all treatments. The methods used for ROM assessment were various: most authors measured it manually, some with the aid of robotic devices or by means of electrical goniometers. ROM assessment at the bedside was not always accurately described among studies, e.g., it was not specified which landmarks were utilized and where the hand goniometer was placed (e.g., medial v. lateral). This might have led to different results. Moreover, some authors did not specify if the evaluation was performed with the knee in a flexed or an extended position, since ROM can greatly differ according to the retraction of the soleus, the gastrocnemius or both muscles of the TS. Finally, some authors measured the whole ROM from maximum plantarflexion to maximum dorsiflexion, according to its definition. Other authors only presented the maximum passive dorsiflexion measured from the neutral position. This discrepancy led to very different raw values among studies. However, improvements in ankle ROM in patients with stroke usually pertain to dorsiflexion measurements alone, so the effect of choosing a different starting point had little value when comparing ROM variations (ΔROM) among studies. It is crucial that future studies write a detailed description of the method used for ROM assessment, to facilitate replicating it and allowing for appropriate meta-analysis.

As outlined by these results, studies have long been emphasizing the need to update spasticity assessment practices for patients who suffered from stroke. The scientific community should foster a debate between experts in the field through topic-specific meetings, such as the Consensus Conferences and the Delphi Panel [91,92]. authors should stop using MAS as the primary outcome of their studies, and journals should similarly refuse studies focused on this outcome. It is now time to transition from outcome measures used out of habit to measures supported by actual evidence.

#### 4.1.2 Methodological considerations on clinical measures

A critical appraisal on the methodologies used in data analysis was conducted.

A total of 39 studies used MAS (or MMAS or CSS) to assess TS spasticity. Nearly half of these studies correctly reported the median score, provided the range, or reported the number of patients of patients for each level of the scale. Afterwards, non-parametric statistics was used to analyze the data (see Tables 4–12). Conversely, in 48% of the studies, authors incorrectly computed mean values and used the t-test (parametric statistics) to compare groups before and after treatment, as if the scales were numerical. MAS is an ordinal scale, i.e., it scores (0, 1, 1+, 2, 3, and 4). Consequently, numbers could just as easily be replaced by letters (e.g., A, B, C, D, E). For this reason, the score cannot be treated as numbers. In MAS scale, scoring a 2 rather than a 1 does not mean that spasticity has increased twofold. Moreover, a 2-point decrease has a different outcome in terms of spasticity reduction, e.g., from 4 to 2, is not the same as from 2 to 0. Treating MAS scores as numbers is misleading and may lead to unreliable conclusions.

When dealing with ordinal scales, computing differences (e.g., ΔMAS), mean values, and comparing groups by parametric statistics, as the t-test, leads to unreliable results and ought to be avoided. On the one hand, the actual effectiveness of a treatment may be not detected. On the other hand, a statistically significant difference between groups could be a result of treatments that are just as effective. For this reasons, non-parametric statistics must be the only one used for ordinal scales like MAS. The same applies for other functional clinicals scales employed in the included studies (e.g., Berg Balance Scale, Fugl-Meyer Assessment, Lower Extremity Functional Scale, Barthel Index, etc.). This methodological error might appear of little consequence to clinicians. However, it is quite the opposite; it is crucial in scientific research since it invalidates the conclusions of many studies published on this topic. This error was present in 22 out of 53 included studies [22,32,35–37,39,40,42–46,48,53,56,59,65–67,70,72,73]. A common incorrect procedure follows: authors compute ΔMAS and the percentage change after treatment (error #1), compare them with the t-test (error #2) and the statistically significant difference of this test is used to state the superiority of the treatment over the placebo (error #3). The rate of reduction can be greater in the experimental group with respect to that of the control group, but the post-intervention confidence intervals of MAS in the two groups can be overlapping, i.e., invalidating the results since there is no real benefit. To guarantee accuracy, journals ought better to consider the inclusion of an expert in methodology among reviewers when reviewing clinical studies.

#### 4.1.3 The instrumental measures of spasticity

Thirty-seven studies (69%) included in this current review combined clinical assessment with instrumental evaluation. Most authors chose a neurophysiological evaluation of the reflex arc, such as H-reflex latency or H/M ratio, especially when delivering shock waves and ultrasound. Direct measurement of the stretch reflex by surface EMG has been used in literature both for children with central palsy [93] and survivors from stroke [94] alike. Fewer studies employed gait analysis and dynamic electromyography to assess the effectiveness of their interventions [31,36,42,69,95].

The ultimate goal of rehabilitation is to recover function, including the ability to walk. Future studies should always consider assessing by how much spasticity limits movement not only at the bedside, but also when walking. It is known that a display of overactivity at the bedside does not always imply the presence of overactivity during gait, because fast joint rotation may be absent [1,96,97]. Many authors are supporting this argument, debating the need to integrate different practices to obtain more accurate information about patient condition [98] and to properly assess overactivity both at rest and while moving [1,96,97,99]. Dynamic instrumental assessment should be considered for patients with stroke [1]. On the one hand, it must be recognized that instrumental evaluation requires resources and specific knowledge to be performed correctly [100–104]. On the other hand, the increasing availability of low-cost wearable devices makes it easier to equip rehabilitation wards with these assessment tools. In addition, universities and the scientific community should make an effort to properly train their students since it is necessary to enable rehabilitation professionals to use these techniques and to correctly interpret data [1,84,95,105].

#### 4.1.4 Future challenges

Few studies included the assessment of stiffness and viscosity, even though these alterations can be present in patients with stroke, along with overactivity [106]. Performing sEMG during gait can help explaining the underlying causes of an alteration in the pattern by differentiating between overactivity and soft tissues modifications. In particular, it would help identifying the main cause in velocity-dependent alterations, between spasticity and increased viscosity, and the main cause in tension-dependent alterations, between spastic dystonia and increased stiffness.

Overlapping patterns from an observational point of view can have completely different causes as described by Campanini et al. [1,105]. The build-up of connective tissue in muscles, as a result of immobilization after a central injury, leads to an increase in muscle stiffness and viscosity [87]. Stiffness and viscosity result in an overall increase in muscle tone. Therefore, with a clinical evaluation, in which the joint is manually mobilized quickly and/or slowly, it is not possible to distinguish when, and how much the response is due to passive and/or reflex components.

In the future, it would be appropriate to routinely introduce sEMG in the clinical assessment to differentiate between the various components of increased muscle tone and consequently choose the appropriate rehabilitation treatment. For example, injection with botulinum toxin can alter the reflex response but not the passive components that are otherwise sensitive to shock waves, dry-needling, stretching, and muscle strengthening [1]. By introducing an integrated evaluation that reveals the underlying causes, it is possible to select a targeted treatment and understand why and when the different treatments reported in literature are effective [3,94,97,107–112].

#### 4.1.5 The issue of taxonomy

The presence in literature of several assessment modalities is a clear indicator of the complexity of the phenomenon being analyzed. Several authors have recently been debating that the word “spasticity” has been misused as an umbrella term to refer as a combination of different central and peripheral phenomena that must instead be considered separately [84]. We agree with the need to differentiate the individual causes underlying the overall phenomenon of EFD between its active, connective-related, and reflex components (see Table 3) [113]. A revision of taxonomy shared among all professionals, such as what happens in the Delphi Panels or at the Consensus Conferences [91,92], is highly advisable. In this way, unclear terms in literature could be avoided, preventing the use of vague and unspecific assessment measures such as MAS. The adoption of a shared language would enable clinicians to choose the most appropriate scales and/or tools according to their needs, to correctly diagnose a patient, and, finally, to select the most appropriate treatment tailored to the patient’s characteristics [97].

### 4.2 Limitations

To our knowledge, this is the first scoping review focusing on the assessment methods for PT treatments of the EVFD with TS spasticity in patients with stroke.

The main limitation of the study is that it only included papers that claimed PT to be able to influence TS spasticity. Since the results highlighted the lack of a common and shared taxonomy in the field of neurorehabilitation, some papers might have been missed due to a different terminology adopted by the authors. Moreover, the search focused on patients with stroke since they represent the main population with acquired neurological disorders often presenting alterations to the lower limbs. The eligibility criteria excluded neurological patients with congenital, degenerative or childhood pathologies because the development of structural soft tissue deformities may follow different patterns. For this reason, the considerations drawn in this review may not be relatable to all studies involving neurological patients, although they could provide an equally interesting insight from a methodological point of view.

## 5 Conclusions

This scoping review summarized all outcome measures and assessment modalities used in literature to assess the effectiveness of PT treatments, when used for the reduction of TS spasticity and EFD in patients with stroke. Clinicians and researchers can find an easy-to-consult synthesis that could be of help to both their clinical and research activities.

The results of this scoping review also highlighted the need to standardize assessment methods employed to evaluate the efficacy of PT interventions on EFD and a gap of knowledge in the appropriate methodology for managing the outcome measures when assessed by ordinal scales. Finally, when PT is the treatment of choice, the use of a shared taxonomy that differentiates the underlying components could lead to identifying the best intervention among those suggested in literature.

## Supporting information

S1 Checklist Preferred Reporting Items for Systematic reviews and Meta-Analyses extension for Scoping Reviews (PRISMA-ScR) checklist. (DOCX)
